# Heritability and genetic correlation between GERD symptoms severity, metabolic syndrome, and inflammation markers in families living in Mexico City

**DOI:** 10.1371/journal.pone.0178815

**Published:** 2017-06-05

**Authors:** Arturo Reding-Bernal, Valentin Sánchez-Pedraza, Hortensia Moreno-Macías, Sergio Sobrino-Cossio, María Elizabeth Tejero-Barrera, Ana Isabel Burguete-García, Mireya León-Hernández, María Fabiola Serratos-Canales, Ravindranath Duggirala, Juan Carlos López-Alvarenga

**Affiliations:** 1 Research Division, Hospital General de México “Dr. Eduardo Liceaga”, Mexico City, Mexico; 2 Endocrinology Service, Hospital General de México “Dr. Eduardo Liceaga”, Mexico City, Mexico; 3 Economy Department, Universidad Autónoma Metropolitana, Mexico City, Mexico; 4 Endoscopy Service, Hospital Ángeles del Pedregal, Mexico City, Mexico; 5 Nutrigenomics Laboratory, Instituto Nacional de Medicina Genómica, Mexico City, Mexico; 6 Department of genetic epidemiology, Instituto Nacional de Salud Pública, Cuernavaca, Morelos, México; 7 South Texas Diabetes and Obesity Institute. University of Texas Rio Grande Valley, Edinburg, TX, United States of America; University Hospital Llandough, UNITED KINGDOM

## Abstract

**Objective:**

The aim of this study was to estimate the heritability (h^2^) and genetic correlation (ρG) between GERD symptoms severity, metabolic syndrome components, and inflammation markers in Mexican families.

**Methods:**

Cross-sectional study which included 32 extended families resident in Mexico City. GERD symptoms severity was assessed by the ReQuest in Practice questionnaire. Heritability and genetic correlation were determined using the Sequential Oligogenic Linkage Analysis Routines software.

**Results:**

585 subjects were included, the mean age was 42 (±16.7) years, 57% were women. The heritability of the severity of some GERD symptoms was h^2^ = 0.27, 0.27, 0.37, and 0.34 (p-value <1.0x10^-5^) for acidity complaints, lower abdominal complaints, sleep disturbances, and total ReQuest score, respectively. Heritability of metabolic syndrome components ranged from 0.40 for fasting plasma glucose to 0.61 for body mass index and diabetes mellitus. The heritability for fibrinogen and C-reactive protein was 0.64 and 0.38, respectively. Statistically significant genetic correlations were found between acidity complaints and fasting plasma glucose (ρG = 0.40); sleep disturbances and fasting plasma glucose (ρG = 0.36); acidity complaints and diabetes mellitus (ρG = 0.49) and between total ReQuest score and fasting plasma glucose (ρG = 0.43). The rest of metabolic syndrome components did not correlate with GERD symptoms.

**Conclusion:**

Genetic factors substantially explain the phenotypic variance of the severity of some GERD symptoms, metabolic syndrome components and inflammation markers. Observed genetic correlations suggest that these phenotypes share common genes. These findings suggest conducting further investigation, as the determination of a linkage analysis in order to identify regions of susceptibility for developing of GERD and metabolic syndrome.

## Introduction

Gastroesophageal Reflux Disease (GERD) and obesity are two of the most common diseases in Western countries. The prevalence of both entities has rapidly increased, which represents a public health problem that involves further analysis. It is estimated that between 20 to 40% of the population in these countries has one or more symptomatic episodes of GERD at least once a month [1–5]. In Mexico, most studies have found consistent figures with those found in other countries, noting that at least one third of the analyzed population has complains of heartburn at least once a month (35–44%) [6,7]. The prevalence of obesity (body mass index, BMI≥30 kg/m^2^) in American population was 33% in 2008 [8]. In Mexico, the prevalence of obesity according to the National Health and Nutrition Survey 2012 was 32% in people over 20 years of age [9].

The relationship between GERD symptoms and obesity remains controversial. However, the results of recent meta-analysis reveal a strong relationship between obesity and GERD [10]. Recently, it has been suggested that abdominal obesity might be an important risk factor for the development of GERD symptoms more than obesity expressed as a high body mass index [11]. Some researchers have formulated the hypothesis that the association between obesity and GERD symptoms, might be due to a physical mechanism because obesity increases the intra-abdominal pressure, leading to an increase in intragastric pressure and consequently, an increase in gradient of gastroesophageal pressure, inducing relaxation of the lower esophageal sphincter [12].

Besides obesity, some studies have suggested an association between gastroesophageal reflux disease and other metabolic syndrome (MS) components, which is defined as a cluster of metabolic disorders such as abdominal obesity, hypertension, hypertriglyceridemia, insulin resistance, hyperglycemia, and low HDL cholesterol concentration [13]. A positive association has been found between the waist circumference (WC) and GERD symptoms [14,15], between hypertension and erosive esophagitis [16,17], between triglyceride concentration and non-erosive GERD [18], between insulin resistance and erosive esophagitis [17,19] and with GERD symptoms scores [17], between hyperglycemia and reflux esophagitis [16] and with GERD symptoms [15], between diabetes mellitus (DM) and abnormal gastroesophageal reflux measured by a 24-hour pH monitoring [20], between MS according to the International Diabetes Federation (IDF) and GERD symptoms [15,17] and with erosive esophagitis [17,19]. Furthermore, a negative association has been found between HDL cholesterol concentration and GERD symptoms scores [14].

Several factors have been identified for the predisposition to develop GERD, such as genetic factors [21,22] that determine the variability in the behavior of gastrointestinal physiology of the upper digestive tract. Some studies have estimated the heritability (h^2^ defined as the proportion of the phenotypic variance attributed to genetic factors) for both GERD symptoms and MS components, but even though recent researches suggest an association between these two entities, there are no studies analyzing the presence of common genetic factors that could share both phenotypes, i.e., a genetic correlation (*ρ*_*G*_) defined as the proportion of the covariance of two phenotypes explained by common genetic factors.

Some studies [21,22] conducted in monozygotic and dizygotic twins, analyzed the heritability of GERD symptoms (heartburn and acid regurgitation), finding that genetic factors considerably contribute to the presence of these symptoms (h^2^ between 0.31 and 0.43). Moreover, other studies [23–28], mostly conducted in families, have estimated the heritability of MS components, finding that a significant proportion of the variability of these phenotypes is explained for genetic factors (h^2^ between 0.25 and 0.9).

The aim of this research was to estimate the heritability of GERD symptom severity, MS components, and inflammation markers, and assess whether these phenotypes share common genes through of genetic correlations (bivariate polygenic equations) in a sample of Mexican families living in Mexico City. This study is the first report of heritability and genetic correlation between GERD symptoms severity and MS components for this population.

## Materials and methods

### Design, study population, and enrollment

Cross-sectional study, in which pamphlets and posters were delivered and posted at universities and public places in Mexico City inviting to participate in this project. This study included 32 extended families living in Mexico City consisting of at least 13 members each, over 18 years of age. The families were selected from a convenience sample and due to the high prevalence of GERD symptoms and MS components in Mexico, no proband or other criteria were considered for inclusion. Potential participants were invited to attend a tertiary level hospital (General Hospital of Mexico “Dr. Eduardo Liceaga”), where researchers, endocrinologists, gastroenterologists, and previously trained nursing staff help with application of questionnaires, obtaining blood samples and anthropometric measurements for each member of the participating families. The enrollment period lasted from September 2010 to September 2014.

Exclusion criteria were those families where one of its members suffered from a genetically transmitted abnormality that could be clinically detected from their clinical history or laboratory tests such as: homozygous or heterozygous familial hypercholesterolemia, hyperchylomicronemia syndrome; hemoglobinopathies; Duchenne’s muscular dystrophy, hemophilia, Huntington’s disease, Marfan syndrome, and phenylketonuria. In addition, pregnant women were excluded due to metabolic changes present during this period, such as greater frequency of GERD symptoms, which could be a potential confounder for the study results. Pregnant women have the highest prevalence of GERD symptoms [29].

### Data-collection instruments and data measurement

#### Questionnaires

Severity of GERD symptoms was assessed by the *ReQuest in Practice* questionnaire consisting of six dimensions (general well-being, acidity complaints, lower abdominal complaints, upper abdominal complaints, nausea, and sleep disturbances) using a visual analog scale of 10 cms, referring to the severity of these symptoms in the last 24 hours. This questionnaire was tried out in Germany, France, Spain, the United Kingdom, and the United States [30,31], and has been translated into different languages to be applied in various locations including Mexico [32,33]. In addition to this questionnaire, participants also filled out a questionnaire of socio-demographic characteristics.

#### Anthropometric and clinical measurements

Measurements for waist circumference (WC), height and weight were taken were taken by previously trained research assistants. WC was measured with a flexible tape at the mid-level between the lower lateral edge of the ribs and the upper side of the iliac crest. This measurement was performed after a normal inhalation and exhalation recorded to the nearest 0.1 cm.

Systolic blood pressure (SBP) and the diastolic blood pressure (DBP) were recorded by a single physician for all members of families, using a sphygmomanometer. The patients should been have resting for 15 minutes before making three independent readings, recording the average of the last two measurements for this study.

#### Biochemical estimations

In the morning, after an overnight fast of at least 8 hours, participants attended the hospital, where they had blood samples extracted for determination of glucose level, high density lipoprotein cholesterol (HDL-C), triglycerides (TG), fibrinogen, and C-reactive protein (CRP) in serum. To determine fasting plasma glucose, the analytical method based on glucose oxidase reaction was used, which has a total coefficient of variation (CV) of 4%. The determination of triglyceride concentration was performed using Trinder enzymatic method, which a total CV of 4.5%. HDL cholesterol concentration was estimated by the method for determining light absorption with a total CV of 4.5%. These biomarkers were determined using LX-20 and LX-20-PRO equipment. Fibrinogen concentration was determined using the optical spectrophotometry technique, which has a total CV of 3.4%, with BCS MAC equipment from Siemens, whilst the concentration of CRP was determined using the nephelometry technique with the 800 IMMAGE equipment from Beckman, with a total CV of 7.5%. The determination of all these markers was undertaken in the central laboratory of the General Hospital of Mexico “Dr. Eduardo Liceaga”.

### Statistical analysis

Continuous variables are reported as the mean ± standard deviation (SD) for age, severity of GERD symptoms (general well-being, acidity complaints, lower abdominal complaints, upper abdominal complaints, nausea, sleep disturbances, and the total ReQuest score indicator defined as the sum of all these symptoms), anthropometric measurements (WC and BMI), metabolic syndrome components (SBP, DBP, concentration of fasting plasma glucose, TG, and HDL-C), and inflammation markers (fibrinogen and CRP). We verified that the variables did not have extreme outliers, that is, that the registers did not exceed the 4 standard deviations in their values. Due to the existence of a sexual dimorphism, a comparison by sex of biochemical and anthropometric variables was performed using the Student’s t-test. For this test, variables for the severity of GERD symptoms were transformed by the square root function, while inflammation and metabolic components were log_10_ transformed where necessary (as determined by the Shapiro-Wilk test). Dichotomous variable of diabetes mellitus (DM) (presence or absence) was reported as the percentage of diabetic people in this population. This variable was compared between men and women using a Pearson’s Chi-square test.

A factor analysis with previously transformed continuous variables was carried out using principal components analysis and a varimax rotation in order to facilitate identification of the variables within each of the factors. The number of factors was determined using the criterion of eigenvalue greater than 1. Variables with loads greater than 0.4 were considered as part of a factor in particular [34]. Statistical tests and factor analysis were performed using STATA software, version 13.0.

The variance-components model partitions the total phenotypic variance of the trait $\left(\sigma_{P}^{2}\right)$, into components that correspond to additive genetic variance $\left(\sigma_{G}^{2}\right)$ and unmeasured environmental variance including non-additive genetic components $\left(\sigma_{E}^{2}\right)$. Given the additive nature of the two components, the estimate of the heritability is given by $h^{2}=\sigma_{G}^{2}/\sigma_{P}^{2}$. That is, the heritability refers to the proportion of phenotypic variance attributed to additive genetic variance. On the other hand, the phenotypic correlation (*ρ*_*P*_) between two phenotypes X and Y is given by $\rho_{P}=\rho_{G}\sqrt{h_{X}^{2}}\sqrt{h_{Y}^{2}}+\rho_{E}\sqrt{\left(1-h_{X}^{2}\right)}\sqrt{\left(1-h_{Y}^{2}\right)}$ where *ρ*_*G*_ and *ρ*_*E*_ are the genetic correlation and the environmental correlation, respectively. $h_{X}^{2}$ and $h_{Y}^{2}$ are the heritabilities of the phenotypes X and Y, respectively. Thus, the genetic correlation (*ρ*_*G*_) is given by $\rho_{G}=\frac{\rho_{P}-\rho_{E}\sqrt{\left(1-h_{X}^{2}\right)}\sqrt{\left(1-h_{Y}^{2}\right)}}{\sqrt{h_{X}^{2}}\sqrt{h_{Y}^{2}}}$, which indicates the association grade between common genetic components. Genetic correlation is the proportion of the covariance of both phenotypes explained by common genetic components [35].

Heritability (*h*^2^) of quantitative traits, was calculated using a standard quantitative genetic variance-components model by the maximum-likelihood method implemented in the Sequential Oligogenic Linkage Analysis Routines (SOLAR) software, version 7.2.5. Before estimating the heritability and genetic correlation of quantitative traits, theses variables were transformed by inverse normal transformation in SOLAR. The residual kurtosis was within normal range after quantitative traits were transformed. The heritability of the diabetes mellitus variable (dichotomous trait) was calculated by a threshold model in SOLAR. The method assumes that an individual belongs to a specific effect status if an underlying genetically determined risk exceeds a certain cut-off point [36]. Except for the heritability of WC and BMI, which were only adjusted for age and sex, all the others phenotypes were adjusted for age, sex and BMI. The estimation of the genetic correlation between the severity of GERD symptoms, MS components and inflammation components was performed with a maximum-likelihood bivariate analysis in SOLAR to find an evidence of shared genetic (or pleiotropy) components, that is, an evidence of additive effects of common genes on the traits. With the exception of the genetic correlation where WC or BMI was involved, which were only adjusted for age and sex, the rest of the genetic correlations were adjusted for the covariables of age, sex, and BMI. The null hypothesis of no genetic effect (*h*^2^ = 0) was assessed. The rejection of *h*^2^ = 0 indicates additive genetic variance contribute to the phenotypic variance in a way statistically significant if *p-value* is <0.05. Heritabilities (*h*^2^) of 0.25 or greater were considered as a significant contribution of the phenotypic variance attributed to additive genetic variance. Tests were performed to test the null hypothesis that *ρ*_*G*_ = 0. The rejection of *ρ*_*G*_ = 0 indicates significant additive effects of common genes on both traits. Evidence of a non-zero estimate for a given correlation was considered statistically significant at *p-value* <0.05. Genetic correlations (*ρ*_*G*_) of 0.2 or greater, whose heritability (*h*^2^) in each of their two involved traits was 0.25 or greater, were considered as a significant contribution of the covariance of both phenotypes explained by common genetic components.

### Ethics statement

This study was approved by the Ethics, Research and Biosecurity Committees from the General Hospital of Mexico “Dr. Eduardo Liceaga”. Each participant received and signed a written informed consent.

## Results

In this study, 32 extended families, resident in Mexico City were included, representing a total of 585 individuals (249 men and 336 women) with a mean age of 41.5 ±16.7 years old. Family size ranged between 13 and 32 individuals with a mean of 16 subjects. The characteristics of age, severity of GERD symptoms, MS components, as well as inflammation components stratified by sex are shown in Table 1. Men showed statistically significant mean values higher than women in waist circumference (93.6 vs. 86.1 cm), triglycerides (199.3 vs. 162.3 ml/dl), while women showed statistically significant higher mean values than men in age (39.3 *vs*. 43.1 years), lower abdominal complaints (1.9 *vs*. 2.4), total ReQuest score indicator (12.0 *vs*. 13.8), HDL cholesterol (37.6 *vs*. 45.7 ml/dl), fibrinogen (294.1 *vs*. 340.1 mg/dl), and CRP (4.2 *vs*. 4.8 mg/dl). Regarding to the percentage of diabetics, women showed a prevalence of nearly six percentage points higher than men (19.1 *vs*. 13.3, p-value = 0.062). No statistically significant differences were found in the mean BMI according to the sex of the participants.

**Table 1:** Description of general data, severity of GERD symptoms, metabolic syndrome components and inflammation components by sex.

Variables	All, n = 585	Men, n = 249	Women, n = 336	p-value^a^
Age (years)	41.5 (16.7)	39.3 (16.9)	43.1 (16.4)	0.006
General well-being of GERD	2.8 (2.2)	2.6 (2.2)	2.9 (2.2)	0.094
Acidity complaints	1.9 (2.3)	1.9 (2.3)	1.9 (2.4)	0.762
Upper abdominal complaints	2.0 (2.3)	1.8 (2.2)	2.2 (2.4)	0.104
Lower abdominal complaints	2.2 (2.5)	1.9 (2.4)	2.4 (2.6)	0.018
Nausea	1.2 (1.8)	1.1 (1.7)	1.2 (1.9)	0.483
Sleep disturbances	3.0 (3.1)	2.6 (2.9)	3.2 (3.2)	0.101
Total ReQuest score	13.0 (10.4)	12.0 (10.4)	13.8 (10.4)	0.018
Waist circumference (cm)	89.3 (13.7)	93.6 (13.7)	86.1 (12.8)	< 0.001
Body mass index (kg/m2)	28.0 (6.1)	28.0 (6.2)	27.9 (5.9)	0.832
Systolic blood pressure (mm Hg)	116.7 (17.1)	117.8 (14.5)	115.8 (18.7)	0.077
Diastolic blood pressure (mm Hg)	73.0 (10.4)	73.9 (10.4)	72.4 (10.4)	0.093
Fasting plasma glucose (mg/dl)	100.3 (39.3)	100.7 (42.9)	100.0 (36.4)	0.926
Triglycerides (mg/dl)	178.0 (117.9)	199.3 (129.8)	162.3 (105.8)	< 0.001
HDL-C (mg/dl)	42.3 (11.2)	37.6 (8.4)	45.7 (11.8)	< 0.001
Fibrinogen (mg/dl)	320.5 (83.0)	294.1 (74.6)	340.1 (83.6)	< 0.001
C-reactive protein (mg/dl)	4.5 (4.6)	4.2 (4.8)	4.8 (4.5)	0.002
Diabetes mellitus (%)	16.6	13.3	19.1	0.062^b^

Data for continuous variables are presented as means (SD). Diabetes mellitus is presented as percentage (%) of diabetics.

^a^ Student's t-test was performed in all continuous variables. The GERD symptom variables were square root transformed, the metabolic syndrome components and inflammation components were log10 transformed.

^b^ Pearson’s chi-square test was calculated.

GERD, gastroesophageal reflux disease; HDL-C, high-density lipoprotein-cholesterol.

After adjusting by age, sex, and BMI, the heritability of the severity of GERD symptoms ranged from 15% (p-value = 0.007) for upper abdominal complaints to 37% (p-value = 1.4 x 10^−8^) for sleep disturbances. Total ReQuest score had a heritability of 34% (p-value = 1.0 x 10^−7^). The continuous variables of the metabolic syndrome presented a heritability which varied from 40% (p-value = 7.2 x 10^−12^) for concentration of fasting plasma glucose to 61% (p-value = 7.2 x 10^−26^) for BMI. DM variable measured as a discrete variable had an h^2^ of 61% (p-value = 1.1 x 10^−5^). Fibrinogen and CRP had an h^2^ of 64 (p-value = 1.0 x 10^−22^) and 38% (p-value = 6.5 x 10^−12^), respectively. Except for the general well-being of GERD, upper abdominal complaints, and nausea, the remainder of the traits had a significant h^2^ greater than 25% (Table 2).

**Table 2:** Heritability estimates (h2) for the severity of GERD symptoms, metabolic syndrome components and inflammation components.

Trait^a^	h^2^ (SE)	p-value	Variation explained by covariates
General well-being of GERD^b^	0.24 (0.08)	4.0 x 10^−5^	-
Acidity complaints^b^	0.27 (0.08)	2.5 x 10^−6^	-
Upper abdominal complaints^b^	0.15 (0.07)	0.007	0.005
Lower abdominal complaints^b^	0.27 (0.08)	7.3 x 10^−6^	0.009
Nausea^b^	0.23 (0.07)	4.2 x 10^−5^	-
Sleep disturbances^b^	0.37 (0.09)	1.4 x 10^−8^	-
Total ReQuest score^b^	0.34 (0.09)	1.0 x 10^−7^	0.009
Waist circumference^c^	0.56 (0.08)	7.5 x 10^−19^	0.16
Body mass index^c^	0.61 (0.07)	7.2 x 10^−26^	0.06
Systolic blood pressure^b^	0.43 (0.07)	3.5 x 10^−16^	0.23
Diastolic blood pressure^b^	0.50 (0.07)	2.0 x 10^−23^	0.14
Fasting plasma glucose^b^	0.40 (0.07)	7.2 x 10^−12^	0.17
Diabetes mellitus^b^^,^^d^	0.61 (0.16)	1.1 x 10^−5^	0.17^e^
Triglycerides^b^	0.40 (0.08)	3.1 x 10^−9^	0.16
HDL-C^b^	0.60 (0.08)	3.5 x 10^−17^	0.21
Fibrinogen^b^	0.64 (0.08)	1.0 x 10^−22^	0.18
C-reactive protein^b^	0.38 (0.08)	6.5 x 10^−12^	0.18

^a^ All traits were treated as continuous except for diabetes mellitus.

^b^ Adjusted for age, sex and BMI.

^c^ Adjusted for age and sex.

^d^ Treated as discrete trait.

^e^ Variation explained by covariates as suggested by the Kullback-Leibler R-squared value.

GERD, gastroesophageal reflux disease; HDL-C, high-density lipoprotein-cholesterol.

Factors and factors loading are shown in Table 3. Four factors were extracted from the 14 continuous traits that were included in the analysis. The first factor (Factor I) explains 24.1% of the total variance, was comprised of the six GERD symptoms from the ReQuest in Practice questionnaire. The MS components were grouped into Factor II and Factor III, formed by the systolic and diastolic blood pressure (14% of the total variance) and waist circumference, fasting plasma glucose, triglycerides and HDL (12.7% of the total variance), respectively. The fourth factor (Factor IV) was formed by fibrinogen and CRP (12.3% of the total variance). After adjusting for age and sex, the estimated heritability for the four factors was 31, 43, 53 and 48%, respectively (P-value < 1.0 x 10^−6^).

**Table 3:** Results of factor analysis, variance components and heritability estimation.

		Factor I	Factor II	Factor III	Factor IV
		(GERD symptoms)	(SBP/ DBP)	(WC/FPG/ TG/HDL-C)	(Fibrinogen/CRP
Factor loadings					
	General well-being of GERD	**0.663**	-0.075	0.055	0.160
	Acidity complaints	**0.805**	0.125	0.077	-0.017
	Upper abdominal complaints	**0.838**	0.034	-0.085	-0.023
	Lower abdominal complaints	**0.823**	0.020	-0.093	-0.012
	Nausea	**0.730**	0.032	-0.009	0.012
	Sleep disturbances	**0.606**	-0.070	0.077	0.083
	WC	0.014	0.380	**0.545**	0.357
	SBP	0.023	**0.901**	0.110	0.068
	DBP	0.049	**0.906**	0.033	0.032
	FPG	0.053	0.210	**0.440**	0.201
	TG	-0.018	0.282	**0.722**	0.085
	HDL-C	0.050	0.068	**-0.837**	0.081
	Fibrinogen	-0.014	-0.020	-0.061	**0.885**
	CRP	0.044	0.147	0.122	**0.844**
Percentaje of variance (%)		24.1	14.0	12.7	12.3
Heritability, h^2^ (SE)		0.31 (0.08)*	0.43 (0.07)*	0.53 (0.08)*	0.48 (0.08)*

All traits were treated as continuous. The GERD symptom variables were square root transformed; metabolic syndrome components and inflammation components were log_10_ transformed.

* Adjusted for age and sex. P-value < 1.0 x 10^−6^

GERD, gastroesophageal reflux disease; WC, waist circumference; SBP, systolic blood pressure; DBP, diastolic blood pressure; TG, triglycerides; HDL-C, high-density lipoprotein-cholesterol; FPG, fasting plasma glucose; CRP, C-reactive protein.

Table 4 shows the results of the analysis of genetic correlation between GERD symptoms, MS components and inflammation markers. The correlations between the severity of the symptoms of GERD and MS components that were clinically relevant and statistically significant were found between: acidity complaints and fasting plasma glucose (*ρ*_*G*_ = 0.40, p-value <0.05); acidity complaints and DM (*ρ*_*G*_ = 0.49, p-value <0.05); sleep disturbances and fasting plasma glucose *ρ*_*G*_ = 0.36, p-value <0.05), and between total ReQuest score and fasting plasma glucose (*ρ*_*G*_ = 0.43, p-value <0.05). Statistically significant correlations between MS components and inflammation markers were found between WC and fibrinogen (*ρ*_*G*_ = 0.42, p-value <0.01); WC and CRP (*ρ*_*G*_ = 0.43, p-value <0.01); BMI and fibrinogen (*ρ*_*G*_ = 0.41, p-value <0.01); BMI and CRP (*ρ*_*G*_ = 0.30, p-value <0.01), and between SBP and fibrinogen (*ρ*_*G*_ = 0.35, p-value <0.05). Genetic correlation between fibrinogen and CRP was *ρ*_*G*_ = 0.42 (p-value <0.01). In all these correlations, the heritability of each of the two traits involved in the polygenic bivariate analysis was greater than 25%.

**Table 4:** Genetic correlation^a^ between severity of GERD symptoms, inflammation components and metabolic syndrome components.

	WC^b^	BMI^b^	SBP	DBP	FPG	DM	TG	HDL-C	Fibrinogen	CRP
General well-being of GERD	0.26 (0.17)	0.21 (0.17)	0.23 (0.19)	0.30 (0.16)	0.39 (0.21)	0.62 (0.27)*	0.04 (0.22)	0.03 (0.19)	0.31 (0.16)	0.25 (0.19)
Acidity complaints	−0.003 (0.17)	−0.09 (0.16)	0.07 (0.18)	0.06 (0.16)	0.40 (0.19)*	0.49 (0.21)*	0.27 (0.20)	−0.08 (0.18)	0.21 (0.16)	0.13 (0.18)
Upper abdominal complaints	−0.18 (0.22)	−0.24 (0.20)	−0.11 (0.22)	0.02 (0.20)	0.48 (0.27)	0.37 (0.33)	0.12 (0.27)	0.001 (0.23)	0.04 (0.21)	0.13 (0.23)
Lower abdominal complaints	0.04 (0.17)	−0.04 (0.16)	−0.004 (0.18)	0.13 (0.16)	0.25 (0.18)	−0.17 (0.22)	−0.13 (0.19)	0.17 (0.18)	−0.22 (0.17)	−0.07 (0.19)
Nausea	−0.13 (0.18)	−0.15 (0.16)	0.01 (0.18)	0.02 (0.16)	0.28 (0.19)	0.01 (0.23)	0.27 (0.21)	0.13 (0.18)	−0.28 (0.18)	−0.04 (0.19)
Sleep disturbances	0.002 (0.16)	0.06 (0.14)	−0.01 (0.16)	−0.05 (0.14)	0.36 (0.17)*	0.23 (0.21)	−0.07 (0.18)	−0.02 (0.16)	−0.12 (0.15)	−0.01 (0.17)
Total ReQuest score	0.02 (0.16)	0.003 (0.15)	0.06 (0.16)	0.09 (0.15)	0.43 (0.18)*	0.33 (0.23)	0.08 (0.19)	−0.04 (0.17)	0.02 (0.16)	0.04 (0.18)
Fibrinogen	0.42 (0.11)**	0.41 (0.11)**	0.35 (0.14)*	0.16 (0.14)	−0.20 (0.15)	0.03 (0.20)	0.28 (0.16)	−0.05 (0.15)	-	0.42 (0.11)**
CRP	0.43 (0.12)**	0.30 (0.11)**	−0.001 (0.13)	−0.15 (0.12)	0.02 (0.13)	−0.01 (0.16)	0.15 (0.15)	0.12 (0.12)	-	-

Data are presented as *ρ*_*G*_(SE).

^a^ All genetic correlations were adjusted for age, sex & BMI except for those that involved WC or BMI.

^b^ The genetic correlation between WC or BMI and another trait was adjusted for age and sex.

* significant genetic correlation at *p < 0.05

** significant genetic correlation **p < 0.01.

GERD, gastroesophageal reflux disease; WC, waist circumference; BMI, body mass index; SBP, systolic blood pressure; DBP, diastolic blood pressure; FPG, fasting plasma glucose; DM, diabetes mellitus; TG, triglycerides; HDL-C, high-density lipoprotein-cholesterol; CRP, C-reactive protein.

## Discussion

In this study, we estimated the heritability of the severity of GERD symptoms, MS components and inflammation markers in a sample of Mexican families, finding that genetic components significantly contribute to the familial segregation of these phenotypes. Regarding to the severity of GERD symptoms, we found that acidity complaints, lower abdominal complaints, sleep disturbances, and the total ReQuest score showed a significant heritability greater than 25%, while the lowest heritability for MS components was 40% for fasting plasma glucose, and the highest was 61% for BMI and DM. Inflammation markers also showed a considerable heritability with 38% and 64% for CRP and fibrinogen, respectively. Concerning the genetic correlation analysis, we found that severity of some GERD symptoms share common genes with some MS components such as acidity complaints with fasting plasma glucose (*ρ*_*G*_ = 0.40) and with DM (*ρ*_*G*_ = 0.49); sleep disturbances with fasting plasma glucose (*ρ*_*G*_ = 0.36); and the total ReQuest score with fasting plasma glucose (*ρ*_*G*_ = 0.43). With respect to MS components and the inflammation markers we found that the traits which share common genes in these families are: fibrinogen with WC (*ρ*_*G*_ = 0.42); fibrinogen with BMI (*ρ*_*G*_ = 0.41); fibrinogen with systolic blood pressure (*ρ*_*G*_ = 0.35); CRP with WC (*ρ*_*G*_ = 0.43), and CRP with BMI (*ρ*_*G*_ = 0.30). Regarding to the inflammation markers, we found that fibrinogen and CRP also share common genes *ρ*_*G*_ = 0.42).

Previous studies in twins have shown an importance of genetic effects for the GERD development, finding a heritability of 43 [21] and 31% [22]. In this study, we also found considerable genetic effects for the severity of GERD symptoms: for total ReQuest score the heritability was 34%. The difference, mainly with the first study, could be because they analyzed the presence of GERD, while we only analyzed the severity of symptoms, in addition, the design of early studies was focused on twins, while we used a family-based design. Due to the fact that several studies [10] have found a positive association between GERD and an increase in BMI, in this study, the heritability was adjusted for age, sex, and BMI, finding that this last variable was not statistically significant in the analysis of the severity of any symptoms, ie, the heritability of the severity of GERD symptoms was not affected by BMI.

Regarding MS components, the heritabilities of continuous traits in this study are in the range of the reported heritabilities in other studies; 37–59% for WC, 16–61% for systolic blood pressure, 12–61% for diastolic blood pressure, 15–59% for fasting plasma glucose, 17–47% for triglycerides, and 54–72% for HDL-C [23,24,26,27]. These differences in heritabilities could be related to the diversity of genetic backgrounds or the level of environmental effects in the population. Moreover, the discrepancy may also be attributed to the variation in the composition of study populations, different sample sizes, covariables included in the analyses, and whether the study is conducted in twins or families. The study by Sung *et al*. [27], based on a study of twins, show the highest heritabilities because twin studies are more likely to show higher heritability estimates compared with those made in family studies [37].

In factor analysis, four clearly differentiated factors were found. Factor 1, was formed of the six GERD symptoms, which was expected due to the construction of the questionnaire ReQuest in Practice [30,31], used to measure the severity of GERD symptoms. The heritability of this component was 31%. The MS components were grouped into two factors: Factor II which was formed of SBP and DBP, and Factor III composed of WC, fasting plasma glucose, triglycerides, and HDL-C. This clustering of metabolic syndrome components coincides with that reported by Lin *et al*. [24] about how factors are formed. Nevertheless, there were differences in the reporting of heritability of them, whereas in our study we found that it was 43 and 53% for Factor II and Factor III, respectively, in the study of Lin *et al*. the heritability was 20 and 44%. In addition to genetic and environmental background, this difference could be due to the formation of pedigrees because in this last study, the family size varied from 3 to 53 members, with an average of 9, while in our study, the formation of pedigrees was more homogeneous with family sizes ranging from 13 to 32 members, with a mean of 16. In another study conducted by Sung *et al*. [27], MS components were also grouped into two factors, but these were formed of lipids and waist circumference for the first factor, and fasting plasma glucose, mean of blood pressure and waist circumference for the second factor. Heritability for these factors was 63 and 60%, respectively. These differences, regarding the findings in our study could be due to Sung *et al*. study Korean population and the design of their study is based on twins. Finally, our Factor IV was formed exclusively of inflammation markers (fibrinogen and CRP), whose heritability was 48%, suggesting that the variability of inflammation components is significantly explained by genetic factors.

The bivariate analysis of polygenic equations shows that the severity of the symptom of acidity shares common genes with the presence of an increased level of fasting plasma glucose and with the presence of DM (*ρ*_*G*_ = 0.40 and *ρ*_*G*_ = 0.49, respectively). These genetic correlations could explain the phenotypic associations found in other studies, such as the association between hyperglycemia and esophagitis for reflux [16], as well as the presence of DM and abnormal gastroesophageal reflux [20]. That is, these phenotypic associations could be explained in large part to the coexistence of common genetic components between these traits. Sleep disturbances and the total ReQuest score also showed a statistically significant genetic correlation with the increasing of fasting plasma glucose. We did not find relevant and statistically significant genetic correlations between the severity of the other GERD symptoms and the other MS components. On the other hand, the statistically significant genetic correlations that we found between the inflammation markers (fibrinogen and CRP), and waist circumference and BMI confirm the strong relationship already established in the literature between these traits, in this case, from the point of view of common genetic components.

One of the limitations that might be pointed out for this study is that we analyzed general or relatively healthy population in terms of the presence of gastroesophageal reflux disease, that is, no proband was considered to determine the presence or absence of this disease. However, it is worth to emphasizing that the aim of this study was to analyze the heritability of the severity of GERD symptoms, not the presence or absence of this pathology. In other words, regardless of the degree of severity of these symptoms, if these are considerably explained by genetic components, it would be expected that, on the whole, the subjects present similar degrees of severity to the rest of the members inside of each family. In this sense, the use of the ReQuest in Practice questionnaire was quite useful due to its quantitative measurement, allowing the estimation of heritability of the severity of GERD symptoms as quantitative traits whose maximization in the heritability analysis is faster and more reliable than the analysis of discrete traits [38]. A strength of this study is the inclusion of extended families, which provide greater statistical efficiency than any other design (nuclear families or twins) for determining heritability, requiring a smaller sample than the other designs [39,40].

In conclusion, in this study we demonstrated that the severity of some GERD symptoms, MS components, and inflammation markers are considerably explained by genetic factors in Mexican families. Statistically significant genetic correlations between the severity of some of GERD symptoms and fasting plasma glucose suggest that the association of genetic components between the severity of GERD symptoms and the metabolic syndrome might be mediated by genetic components involved in the level of plasma glucose concentration. These findings provide evidence that justifies conducting further investigation, as the determination of a linkage analysis in order to identify regions of susceptibility for developing of GERD and metabolic syndrome.

## Supporting information

S1 Dataset All initial variables with which the presented information in this manuscript was obtained. (XLSX)
